# Provider Fatigue During Direct Manual Compression for Life-Threatening Bleeding

**DOI:** 10.7759/cureus.17487

**Published:** 2021-08-27

**Authors:** Nathan Charlton, Keke Schuler, Chi H Ho, James Hatten, William A Woods, Craig Goolsby

**Affiliations:** 1 Emergency Medicine, University of Virginia, Charlottesville, USA; 2 Emergency Medicine, National Center for Disaster Medicine and Public Health, Bethesda, USA; 3 Public Health Sciences, University of Virginia, Charlottesville, USA; 4 Military & Emergency Medicine, Uniformed Services University of the Health Sciences, Bethesda, USA

**Keywords:** hemorrhage, hemorrhagic shock, first aid, pressure, traumatic shock, penetrating wounds

## Abstract

Introduction

Trauma is a leading cause of death throughout the world, with hemorrhage being responsible for more than 35% of pre-hospital trauma deaths and more than 40% of deaths within the first 24 hours after injury. Despite first aid having a demonstrable effect on mortality from trauma, relatively little research has compared the best methods for bleeding control in the prehospital first aid setting. The most common first-line therapy for external bleeding control in the pre-hospital first aid setting is direct manual compression (DMC). However, a prior study demonstrated that the primary cause of failure in a simulated model of life-threatening bleeding was the inability to maintain adequate direct pressure for three minutes. In this study, we evaluated the effect of fatigue on DMC for the duration of a typical urban emergency medical services (EMS) response time.

Methods

We conducted a prospective observational trial of 33 participants, 18 years of age or older to measure the pressure generated on a model of life-threatening bleeding over an eight-minute period using a “CPR posture” for applying pressure. The primary analyses were longitudinal two-level multilevel models (MLM) with repeated measures of outcome (i.e., CPR posture pressure) nesting within participants. The demographic factors of gender, age, and weight were included as moderators in the analyses and each was analyzed independently.

Results

The participants’ average age was 31 (SD = 11) and the average weight was 161 pounds (SD = 31). The sample consisted of 18 female participants (54.5%) and 15 male participants (45.5%). Applied DMC pressure declined over time, more sharply initially from the beginning to approximately 250 seconds, at which point the decrease in pressure was gradual. Of the demographic factors, gender was associated with a difference in cardiopulmonary resuscitation (CPR) posture pressure over time.

Conclusion

Rescuers should be aware that fatigue may occur and may affect the quality of direct manual compression for control of life-threatening bleeding. Further research is needed to define the external pressures needed to control life-threatening bleeding and the extent that rescuer fatigue affects this pressure.

## Introduction

Trauma is a leading cause of death throughout the world [1]. Hemorrhage is a major cause of trauma-related mortality causing more than 35% of pre-hospital trauma deaths and more than 40% of deaths within the first 24 hours after injury [2]. First aid has been demonstrated to improve survival from trauma and may be of increased importance in low- to middle-income countries without well-established trauma systems [3]. Despite first aid having a demonstrable effect on mortality from trauma, relatively little research has compared the best methods for bleeding control in the prehospital first aid setting [4].

The most common first-line therapy for external bleeding control in the pre-hospital first aid setting is direct manual compression (DMC) [4,5]. DMC is readily available as it can be performed by laypeople and does not require any equipment. While evidence suggests that manufactured tourniquets should be the first-line therapy for life-threatening extremity bleeding, a manufactured tourniquet may not always be immediately available, and therefore DMC should be used while awaiting the application of a tourniquet [5,6]. In conjunction with a hemostatic dressing, DMC is the mainstay therapy for life-threatening head, neck, junctional, and truncal bleeding in the pre-hospital setting [5,6].

The treatment of life-threatening external bleeding requires the provider to exert significant force to compress injured vessels, slow bleeding, and promote clotting. Prior data suggest that a “CPR posture” using two interlocking hands with extended arms and body weight force is superior to other body positions used to deliver DMC [7, 8]. However, a study evaluating laypeople’s use of hemostatic dressings demonstrated that the primary cause of failure in a simulated model of life-threatening bleeding was the inability to maintain adequate direct pressure for three minutes [9]. Prior research demonstrates that in another manual life-saving technique, chest compressions during cardiopulmonary resuscitation (CPR), quality degrades over time due to rescuer fatigue [10-13]. The impact of fatigue on laypeople’s application of DMC for life-threatening bleeding is not known. The purpose of this study was to evaluate the effect of fatigue on DMC for the duration of a typical urban emergency medical services (EMS) response time.

## Materials and methods

A prospective observational trial was conducted to measure the force generated over time by DMC using a “CPR posture” on a simulator. A convenience sample of 33 participants was enrolled. The inclusion criteria were that participants were 18-years old or older and that the participant must willingly enroll after hearing the physical demands of the study procedure. After obtaining verbal consent, participant demographics including self-reported height, weight, and gender were recorded. Information about prior first aid or medical experience, as well as injuries that might affect performance was collected. Participants received a brief training on two-handed DMC using the “CPR posture” by the researchers. Participants were then asked to kneel beside the simulator and apply pressure to the simulator using a CPR posture.

**Figure 1:**
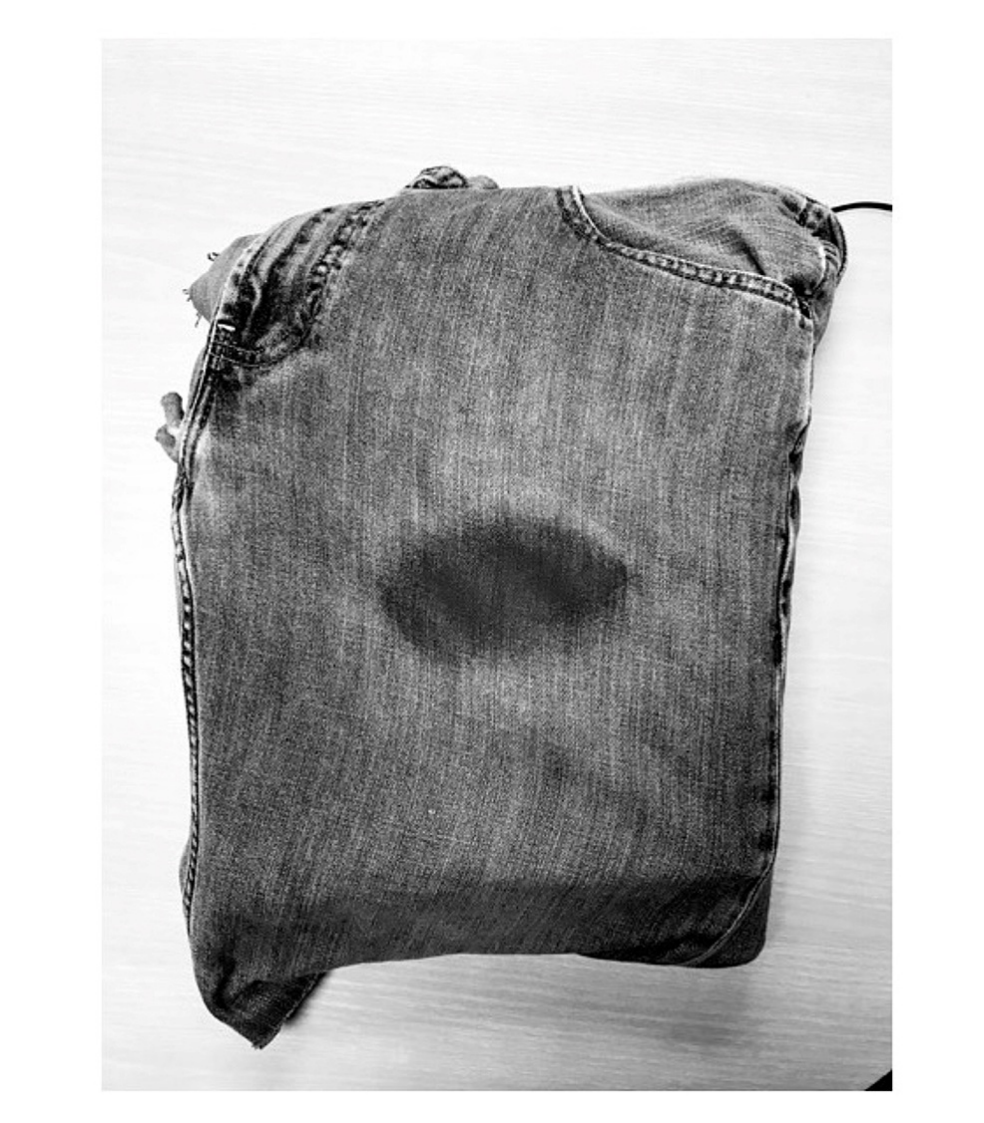
Bleeding control model.

The bleeding control model used resembled an inguinal area with external bleeding. Clothing, consisting of blue jeans, was externally stained with simulated blood in the inguinal area (Figure 1). This model covered a force plate (Vernier, Beaverton, OR, US) which was used in the model to record the amount of force generated on the model over time. Output was recorded during the study period every two seconds as pounds per square inch (psi) using Vernier Graphical Analysis software v4.10.0 (Beaverton OR, US). 

The experimental design required each participant to hold pressure for eight minutes. In an effort to reduce bias, participants were not told the total time that they would be applying pressure. The participants were instructed to hold pressure while awaiting EMS arrival, and that the total time of their participation would be less than 15 minutes. Participants were also not updated on elapsed time once they began applying pressure. A duration of eight minutes was selected to mimic the average urban EMS response time (14). Participants were asked to inform the researchers when they felt fatigue affecting their performance. Participants could stop if they were in discomfort or too fatigued to continue. Participants received a $5 gift card as compensation for their time. The University of Virginia IRB reviewed and approved this protocol (IRB-HSR-21315).

Data analysis

The primary analyses were longitudinal two-level multilevel models (MLM) with repeated measures of outcome (i.e., CPR posture force) nesting within participants. MLM analyses were performed with two effects, a linear effect and a quadratic effect, which allowed us to capture potential non-linear change in the CPR posture pressure over time. To further assess the effects of demographic factors on CPR posture, force over time, gender, age, and weight were included as moderators in the analyses and each were analyzed independently. The MLM analyses were performed in R using “lme” function in the “nlme” package, with slopes and intercepts varying between participants. To compare the linear model and the non-linear model, model fit information, Akaike information criterion (AIC), and Bayesian information criterion (BIC) were used. Generally, a model with smaller AIC and BIC values is considered to have a better model fit.

## Results

The participants’ average age was 31 (SD = 11) and the average weight was 161 pounds (SD = 31). The sample consisted of 18 female participants (54.5%) and 15 male participants (45.5%).

**Figure 2:**
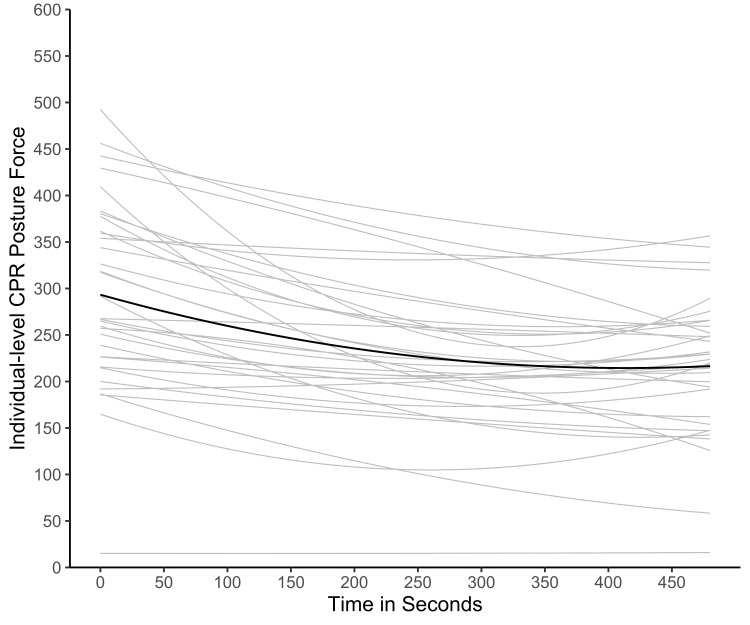
CPR posture pressure over time. CPR: cardiopulmonary resuscitation.

Results for CPR posture pressure change over time are summarized in Table 1. The non-linear model was significant and showed better model fit than the linear model, indicating participants’ applied CPR posture pressure declined more sharply initially from the beginning to approximately 250 seconds. From the 250 seconds point on, the decrease in CPR posture pressure was gradual (Figure 2). These results were consistent with the timing of participants’ self-reported fatigue, which occurred on average at 237 (SD = 92) seconds.

**Table 1:** CPR posture pressure change over time. AIC: Akaike information criterion; BIC: Bayesian information criterion; Time^2^: Time squared; CPR: cardiopulmonary resuscitation.

	Linear Model		Nonlinear Model	
	Coefficient	p	Coefficient	p
Intercept	276.8	< .001	293.3	< .001
Time	-0.2	< .001	-0.4	< .001
Time^2^	-	-	0.0005	< .001
AIC	76276.5		75409.4	
BIC	76318.2		75478.9	

**Figure 3:**
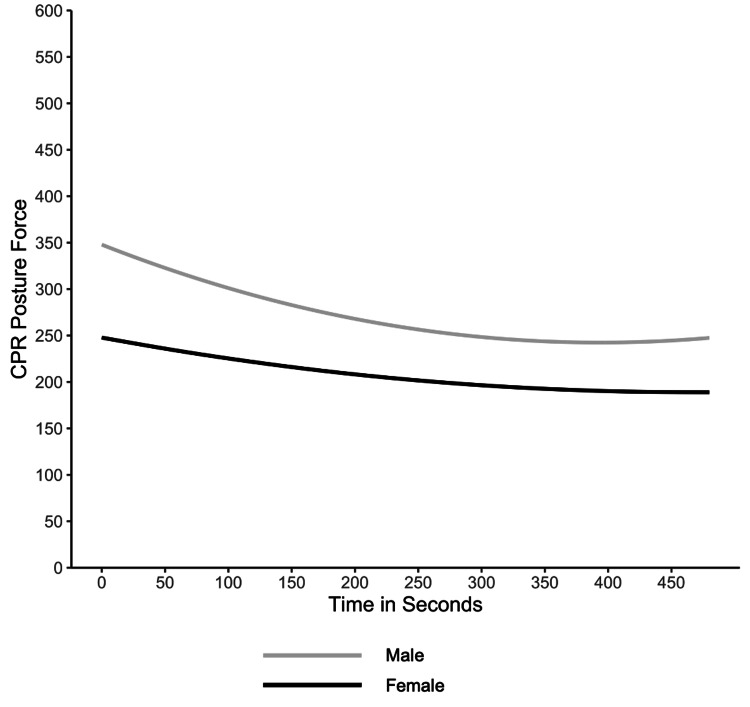
CPR posture pressure over time by gender. CPR: cardiopulmonary resuscitation.

Demographic factors recorded included prior experience, pre-existing medical conditions, age, weight, and gender. Only one significant factor emerged: gender, suggesting a gender difference in CPR posture pressure over time (Table 2). Specifically, the initial decline of CPR posture pressure was steeper for male participants than for female participants, and male participants applied higher CPR posture pressure overall. (Figure 3).

**Table 2:** CPR posture pressure over time by gender. ^a^Moderator represents gender, age, and weight, respectively, in each corresponding model. Time^2^ = Time squared; CPR: cardiopulmonary resuscitation.

	Gender		Age		Weight	
	Coefficient	p	Coefficient	p	Coefficient	p
Intercept	247.8	< .001	318.2	< .001	-63.7	0.355
Time	-0.3	< .001	-0.6	0.001	0.0	0.891
Time^2^	0.0	.032	0.0	0.014	-0.0	0.981
Moderator^a^	100.1	.003	-0.7	0.661	2.2	< .001
Time Interaction	-0.3	.012	0.0	0.267	-0.0	0.171
Time^2^ Interaction	0.0	.032	0.0	0.335	0.0	0.364

## Discussion

In this study conducted on a model of life-threatening bleeding, a decrease in pressure generated over time during the eight-minute time period was observed. This suggests that fatigue does play a role in rescuer ability to generate pressure to stop life-threatening bleeding. Gender was the only demographic factor that affected fatigue in a statistically significant manner.

Fatigue is a factor that may play a role in the efficacy of manual rescue techniques. Studies evaluating fatigue in cardiopulmonary resuscitation provide insight into the topic of rescuer fatigue [10-13]. One study assessing two different CPR methods found that compression-only CPR had a higher number of adequate chest compressions than standard CPR during the first two minutes [14]. Multiple other studies have demonstrated that the percentage of adequate chest compressions that a single rescuer provides diminishes over time [10,12,13]. In addition, when performing CPR, rescuers may fail to recognize when fatigue affects performance, resulting in unintended harm from a fatigued rescuer [12]. 

This study demonstrated that fatigue occurs over time, however, based on available data, there was not a specific inflection point where the pressure applied markedly diminished. Participants reported feeling that fatigue affected their performance about halfway through the study (mean 237 seconds), and there was a corresponding decrease in the slope of pressure decay around this time as well (250 seconds). It is possible that self-perceived rescuer fatigue is a more adequate reflection of performance than in CPR. This study was limited to eight minutes to mimic an average urban EMS response time. For rural and wilderness areas which may have considerably longer EMS response times, fatigue may play more of a factor. While pressure generated on the force plate in this study often exceeded an average adult systolic blood pressure, the specific pressure needed to stop life-threatening bleeding as would be measured at the body surface at the site of hemorrhage remains unknown and likely varies based on patient factors. As more data become available regarding the pressures needed to stop life-threatening bleeding, data from the study may be useful in determining the time period in which pressure applied by a single rescuer using the CPR method is most effective. 

A prior study found that a two-handed method of hemorrhage control generated more pressure than either opposing hands or a single-hand method [7]. However, it must be recognized that not all areas of the body may be accessible to pressure applied in this manner. These areas potentially include the neck, axillae and areas of the hip or abdomen. In these cases, an opposing hand method could be used [7,8]. Using opposing hand to apply force generates a pressure that is almost equivalent to the two-hand methods; however, it is not currently known how fatigue affects this opposing hand method [8]. It is possible that alternative methods to deliver pressure, such as applying force with a knee, may be less susceptible to fatigue [15].

A prior study evaluating the use of hemostatic dressings by laypeople demonstrated that the most common cause of unsuccessful dressing application was failure to apply pressure greater than 250 mmHg for the required amount of time [9]. Hemostatic dressings, recommended as adjunctive therapy to DMC, may decrease the time and pressure needed for DMC to stop life-threatening bleeding, helping to overcome the issue of rescuer fatigue [6,16]. In addition, Goolsby et al demonstrated that a hemostatic dressing that does not rely on pressure applied by a rescuer resulted in fewer application errors compared to those dressings that require a rescuer to apply manual compression [9]. 

Limitations

This study has multiple limitations that should be addressed. This study was conducted in a controlled setting with a model of bleeding control, and rescuer applied pressure and resultant fatigue may vary in real-life situations where presence of an audience or adrenaline may affect performance. In addition, only adult participants were enrolled and the minimum reported bodyweight of the participants was 113 pounds. It is possible that individuals with a lower body weight than the study participants may be less likely to exert pressure in excess of systolic blood pressure and may be more likely to experience fatigue. Therefore, it is possible that children or small adults may not exert enough force to stop life-threatening bleeding. Currently, the external pressures needed to halt life-threatening bleeding are unknown and may vary based on location of the bleed, size of the individual and blood pressure of the individual. Until there is further understanding of these factors, it is unknown how the data in the study translate into the real-world setting. 

## Conclusions

To better assess the ability of first aid providers to use direct manual compression to stop a life-threatening bleed, this study was conducted to evaluate the effect of fatigue over time when applying direct manual compression to a bleeding control model. This study demonstrated that the pressure exerted by direct manual compression pressure decreased over the eight-minute study period when tested on a bleeding control model. Gender was the only statistically significant factor that affected pressure applied over time. When treating life-threatening bleeding, rescuers should be aware that fatigue may occur and may affect the quality of direct manual compression for control of life-threatening bleeding. However, further research is needed to define the external pressures needed to control life-threatening bleeding and the extent that rescuer fatigue affects this pressure.
